# Zinc-Chelation Contributes to the Anti-Angiogenic Effect of Ellagic Acid on Inhibiting MMP-2 Activity, Cell Migration and Tube Formation

**DOI:** 10.1371/journal.pone.0018986

**Published:** 2011-05-04

**Authors:** Sheng-Teng Huang, Rong-Chi Yang, Hsiao-Ting Wu, Chao-Nin Wang, Jong-Hwei S. Pang

**Affiliations:** 1 Department of Chinese Medicine, Chang Gung Memorial Hospital - Kaohsiung Medical Center, Chang Gung University College of Medicine, Kaohsiung, Taiwan; 2 Chinese Herbal Pharmacy, Chang Gung Memorial Hospital, Tao-Yuan, Taiwan; 3 Department of Obstetrics and Gynecology, Chang Gung Memorial Hospital, Tao-Yuan, Taiwan; 4 Graduate Institute of Clinical Medical Sciences, Chang Gung University, Tao-Yuan, Taiwan; Enzo Life Sciences, Inc., United States of America

## Abstract

**Background:**

Ellagic acid (EA), a dietary polyphenolic compound, has been demonstrated to exert anti-angiogenic effect but the detailed mechanism is not yet fully understood. The aim of this study was to investigate whether the zinc chelating activity of EA contributed to its anti-angiogenic effect.

**Methods and Principal Findings:**

The matrix metalloproteinases-2 (MMP-2) activity, a zinc-required reaction, was directly inhibited by EA as examined by gelatin zymography, which was reversed dose-dependently by adding zinc chloride. In addition, EA was demonstrated to inhibit the secretion of MMP-2 from human umbilical vein endothelial cells (HUVECs) as analyzed by Western blot method, which was also reversed by the addition of zinc chloride. Reversion-inducing cysteine-rich protein with Kazal motifs (RECK), known to down-regulate the MMP-2 activity, was induced by EA at both the mRNA and protein levels which was correlated well with the inhibition of MMP-2 activity. Interestingly, zinc chloride could also abolish the increase of EA-induced RECK expression. The anti-angiogenic effect of EA was further confirmed to inhibit matrix-induced tube formation of endothelial cells. The migration of endothelial cells as analyzed by transwell filter assay was suppressed markedly by EA dose-dependently as well. Zinc chloride could reverse these two effects of EA also in a dose-dependent manner. Since magnesium chloride or calcium chloride could not reverse the inhibitory effect of EA, zinc was found to be involved in tube formation and migration of vascular endothelial cells.

**Conclusions/Significance:**

Together these results demonstrated that the zinc chelation of EA is involved in its anti-angiogenic effects by inhibiting MMP-2 activity, tube formation and cell migration of vascular endothelial cells. The role of zinc was confirmed to be important in the process of angiogenesis.

## Introduction

Ellagic acid (EA) is a dietary polyphenol known to be present abundantly in fruits and vegetables [1]–[3]. The multiple effects of EA such as antioxidant, anti-proliferative, chemopreventive, and anti-atherogenic properties have been demonstrated in different studies [1]–[9]. EA exerts its effects via activation of various signaling pathways, including apoptosis, protection from oxidative DNA damage or LDL-oxidation, and alteration of growth factor expression, as well as through the expression of p53, NF-kappa B, and peroxisome proliferator-activated receptor (PPAR) family responsive genes [4], [5], [7], [8], [10], [11]. However, only a few studies regarding the anti-angiogenic effect of ellagic acid have been reported. EA has been reported to act as a potent nucleoside diphosphate kinase (NDPK-B) inhibitor and potentially might reduce the local ATP levels and P2Y receptor-mediated angiogenesis [12]. EA has also been demonstrated to inhibit vascular endothelial growth factor (VEGF)-induced migration of endothelial cells as well as their differentiation into capillary-like tubular structures, and abolish platelet-derived growth factor (PDGF) dependent smooth muscle cell migration [13]. The more detailed mechanism responsible for the anti-angiogenic effect of EA is still needed to be further explored.

Angiogenesis is known to play an important role in cancer development for oxygen and nutrient supply [14]. Tumor cells produce angiogenic factors including bFGF, VEGF and PDGF to promote growth of the tumor. These angiogenic factors increase endothelial cell permeability, migration, invasion and stabilization of capillary tubes that are associated with the expression of matrix metalloproteinases (MMPs), a family of zinc- and calcium-dependent enzymes [15]. The first step in the angiogenic process is the degradation of subendothelial basement membrane and surrounding extracellular matrix [16]. Following matrix breakdown, endothelial cells can migrate and proliferate to form new blood vessels. The matrix metalloproteinases (MMPs) are highly expressed in cells that are involved in angiogenesis both in vitro [17] and in vivo [18]. Inhibition of the early degradation of extracellular matrix predominantly by MMPs is considered an important strategy for anti-angiogenesis. MMP-2 and MMP-9 have been considered as enzymes in degradation of the stroma and extracellular basement proteins to allow further differentiation and spread of endothelial cells during angiogenesis. Reversion-inducing cysteine-rich protein with *K*azal motifs (RECK) protein is known to inhibit MMP-2 and MMP-9 activities and lead to strong suppression of invasion, metastasis, and tumor angiogenesis [19], [20]. Therefore, the induction or over expression of RECK by drug may be considered as an anti-angiogenic or anti-cancer approach.

We previously reported that EA could inhibit in vivo angiogenesis as determined by embryo chorioallantoic membrane (CAM) vascular growth assays which was correlated well with the direct inhibition of MMP-2 activity and the secretion of MMP-2 from human vascular endothelial cells [21]. EA has been shown to acquire the ability to bind zinc ion and form metal-complex [22], [23]. In addition, zinc is known to act as a catalytic ion critical for the enzymatic activity of MMP-2 protein. Therefore, in the present study, we aimed to investigate whether the zinc chelating activity of EA contributed to its anti-angiogenic effect.

## Results

### Zinc chloride reversed the direct inhibition of MMP-2 activity by ellagic acid

**Figure 1:**
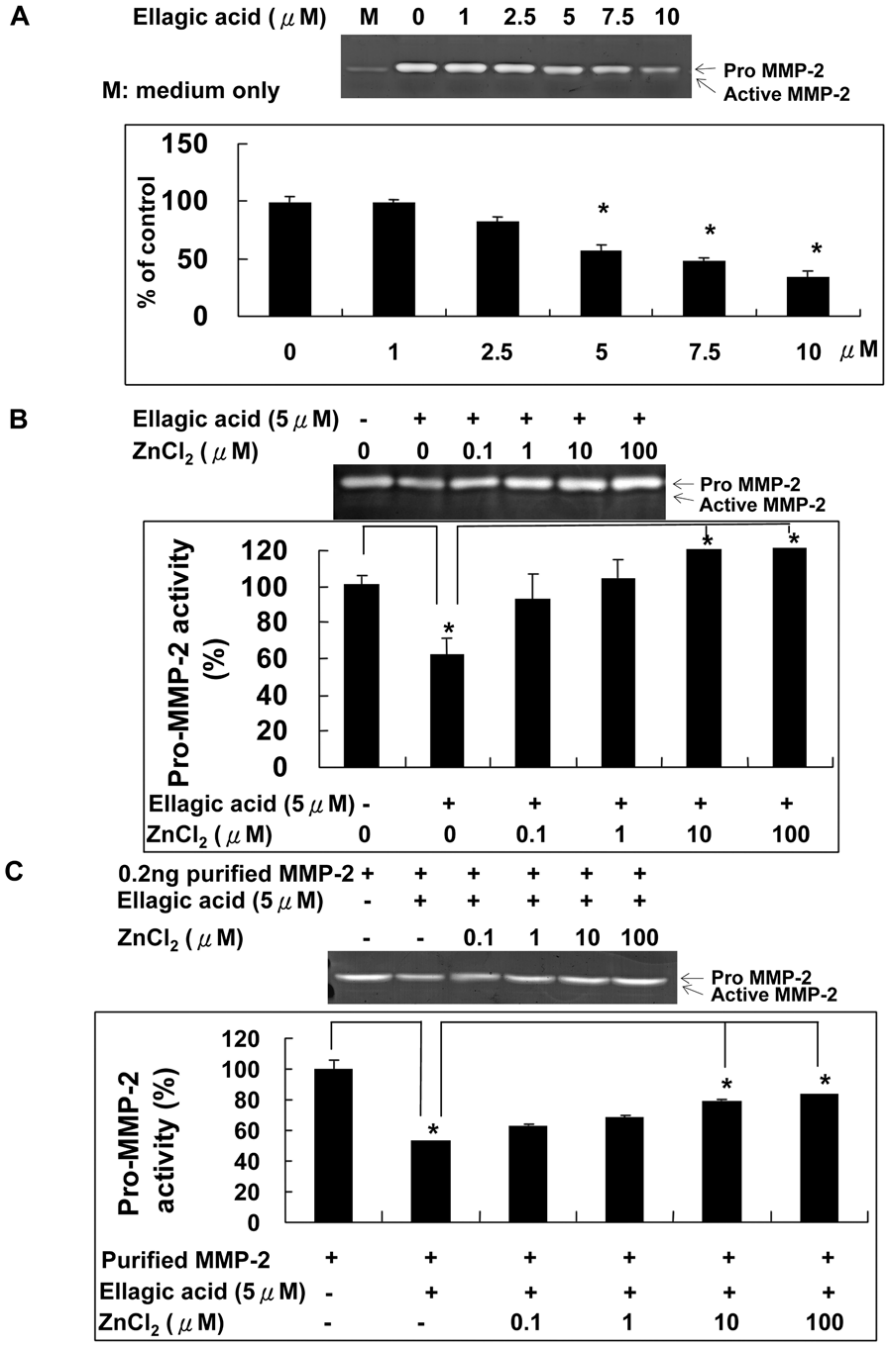
Zinc chloride reversed the direct inhibition of MMP-2 activity by ellagic acid. A. EA directly inhibited the MMP-2 activity in cell-free conditioned medium dose-dependently. B. Co-incubation with ZnCl_2_ reversed the direct inhibition of MMP-2 activity in cell-free conditioned medium by EA. Conditioned medium containing the MMP-2 activity was taken from cultures of HUVECs. C. The activity of purified MMP-2 in the presence of EA was inhibited which could also be reversed by ZnCl_2_ dose-dependently.

As reported in our previous study [21], EA could inhibit the MMP-2 activity both in vivo and in vitro. Proteolytic degradation of ECM components by MMP-2 is demonstrated critical for angiogenesis. The inhibition of MMP-2 therefore contributes to the anti-angiogenic effect of EA. Since EA is able to complex with zinc ion and MMP-2 is a zinc-dependent enzyme, we first examined the effect of EA directly on MMP-2 activity. As shown in Fig. 1A, the direct incubation of conditioned medium containing MMP-2 protein with ellagic acid dose-dependently decreased the MMP-2 activity as measured by gelatin zymography. By adding zinc chloride together with EA to condition medium reversed MMP-2 activity inhibited by EA in a dose-dependent manner (Fig. 1B). Similar results were obtained when purified MMP-2 protein alone was incubated with EA directly (Fig. 1C). We found that the MMP-2 activity was inhibited and this inhibitory effect of EA could also be reversed by adding ZnCl_2_. This result strongly suggested a direct binding between EA and MMP-2 and the binding was Zn-dependent.

### Direct binding of EA to MMP-2 protein was confirmed by change of UV absorption

**Figure 2:**
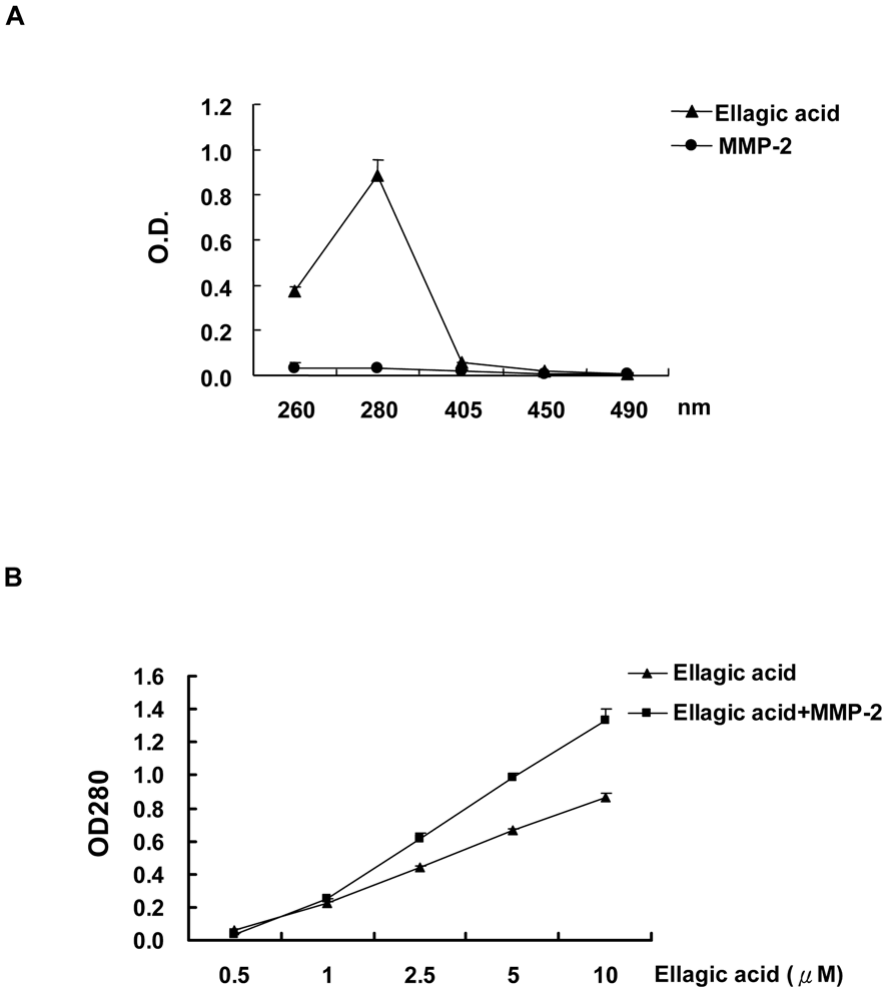
Ellagic acid directly bound to MMP-2 protein as demonstrated by the change of UV absorption. A. The spectral absorptions of 10 µM EA and 0.2 ng MMP-2 were measured at 260, 280, 405, 450 and 490 nm. B. The spectral absorptions at 280 nm for EA ranged from 0.5–10 µM in the absence or presence of MMP-2 protein were measured.

Most drugs have distinct UV or visible spectra because of the conjugated chromophores in the molecule. When a drug interacts with a protein the spectrum may be changed because of alterations in the electronic configuration which can be used to analyze the drug-protein interaction. As shown in figure 2A, the maximum UV absorption for 10 µM EA was determined to be 0.886±0.0684 at 280 nm. The OD_280_ for MMP-2 was as low as 0.031±0.0015. In Fig. 2B, the absorption of EA ranged from 0.5–10 uM at OD_280_ was from 0.0640±0079 to 0.863±0.0718, respectively. However, in the presence of MMP-2, the absorption of EA was increased dramatically to 1.328±0.0247 when EA was 10 µM. Since the OD_280_ for MMP-2 protein was extremely low,, the increased UV absorption was simply due to the change of EA configuration after binding to MMP-2 protein.

### Zinc chloride reversed the inhibition of MMP-2 activity by ellagic acid in human endothelial cells

**Figure 3:**
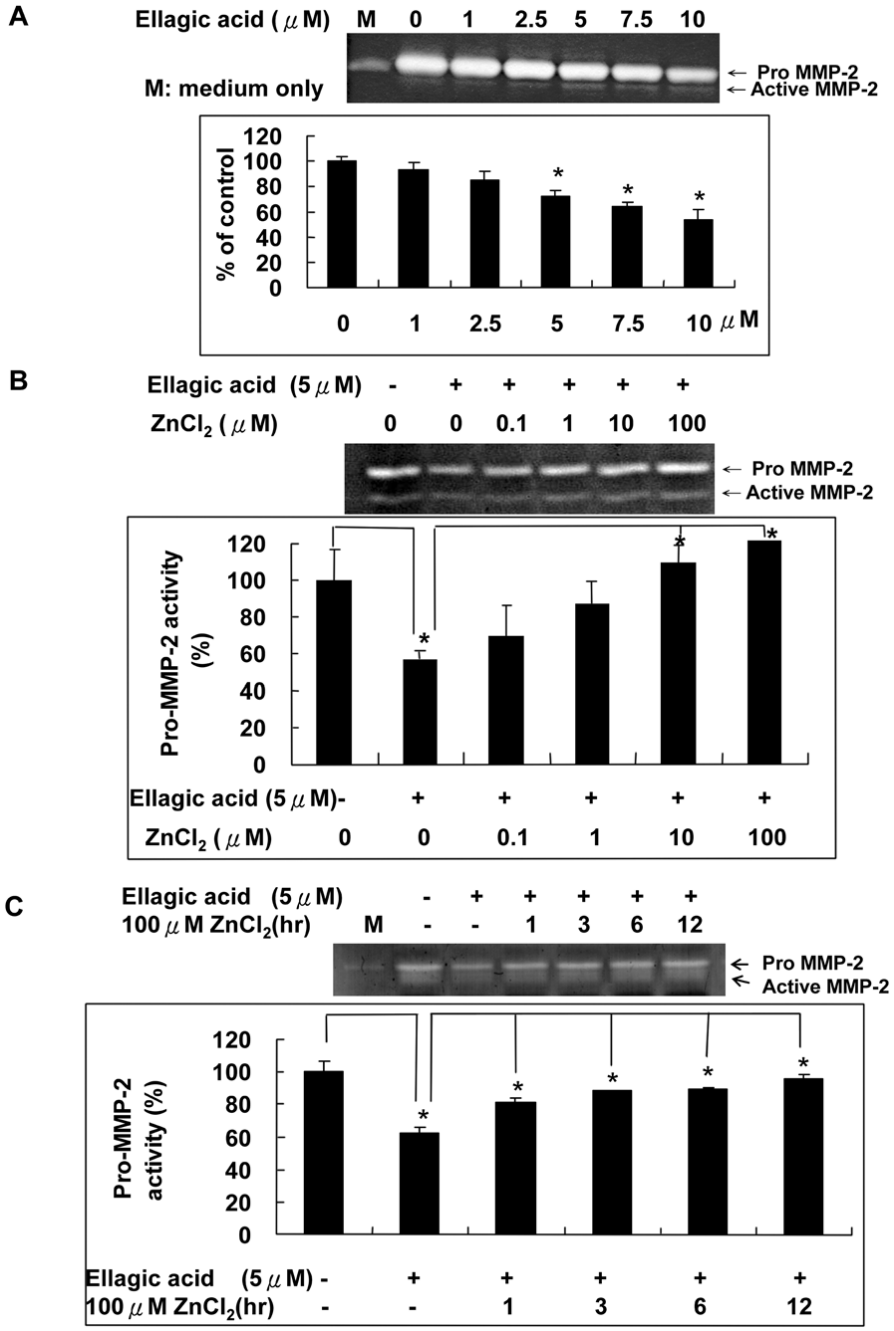
Zinc chloride reversed the inhibition of MMP-2 activity by ellagic acid in human endothelial cells. A. EA dose-dependently inhibited the MMP-2 activity of HUVECs. B. Co-incubation of 5 µM EA with ZnCl_2_ ranged from 0–100 µM for 24 hours reversed the inhibition of MMP-2 activity of HUVECs by EA. C. HUVECs were treated first with 5 µM EA alone and the culture media were replaced at 1, 3, 6 and 12 hours later by fresh culture media containing 100 µM ZnCl_2_. Conditioned media were collected 24 hours later and used for the gelatin zymography analysis.

To further study whether the effect of EA on MMP-2 activity may also go through a cellular mechanism in HUVECs, we examined the MMP-2 activity in HUVECs with EA treatment for 24 hours. Results revealed that the MMP-2 activity in the conditioned medium collected from EA-treated cells for 24 hours was dose-dependently inhibited (Fig. 3A). By adding ZnCl_2_, at concentrations ranging from 0.1 to 100 µM, the MMP-2 activity could be dose-dependently restored (Fig. 3B). MTT assay was performed and the results indicated that higher concentration of EA could cause the decrease of cell number due to the inhibition of cell proliferation, but not cell death. Since the the percentage of cell number compared to control after EA treatment was found to be 98±6%, 98±5%, 92±2%, 82±2% and 76±0% for EA at 1, 2.5, 5, 7.5 and 10 µM, respectively, we decided to use 5 µM for the experiments. We also performed the experiment by treating vascular endothelial cells first with EA alone. The media were then replaced at 1, 3, 6 and 12 hours later by fresh media containing ZnCl_2_ and conditioned media were collected at 24 hours for zymography analysis. The result shown in Fig. 3C demonstrated that the addition of ZnCl_2_ even at 12 hours after the entrance of EA into cytoplasm could partially reverse the inhibitory effect of EA, indicating a Zn-dependent intercellular pathway is also involved in the regulation of MMP-2 activity in HUVECs.

### Zinc chloride reversed the inhibition of MMP-2 secretion by ellagic acid

**Figure 4:**
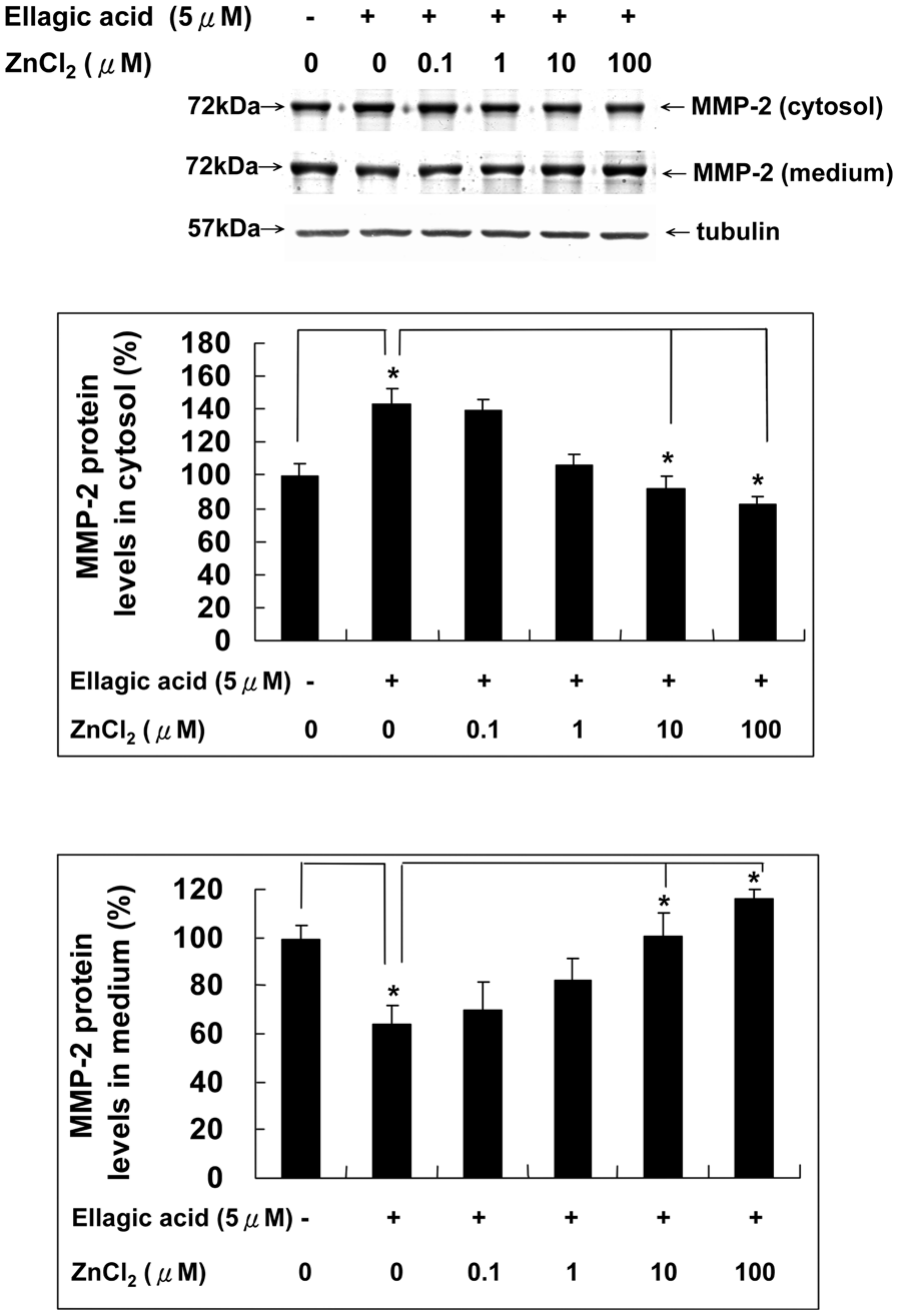
Zinc chloride reversed the inhibition of MMP-2 secretion by ellagic acid. Cells treated without or with EA in the presence or absence of ZnCl_2_ for 24 hours were processed for Western blot method to analyze the protein levels of MMP-2 in conditioned media and in cytosol.

The process of MMP-2 secretion to the extracellular environment where it actually functions could be another critical mechanism for the regulation of angiogenesis. In our previous study [21], we demonstrated that the protein levels of MMP-2 in conditioned medium after EA treatment were dose-dependently decreased and the protein levels of MMP-2 in the cytosol were conversely increased after EA treatment in a dose-dependent manner. The accumulation of MMP-2 protein in the cytosol and the decrease of MMP-2 protein in conditioned medium indicated that a cellular mechanism was involved in the inhibited secretory pathway of MMP-2 by EA. We further investigated whether the presence of ZnCl_2_ could affect this EA effect. As shown in Fig. 4, the decreased MMP-2 level in conditioned medium and the increased MMP-2 level in cytosol of EA-treated HUVECs were gradually reversed by co-incubation with ZnCl_2_. ZnCl_2_ at 10 µM totally abolished the inhibitory effect of EA on the secretion of MMP-2 in HUVECs.

### Ellagic acid enhanced the RECK expression suppressed by zinc chloride

**Figure 5:**
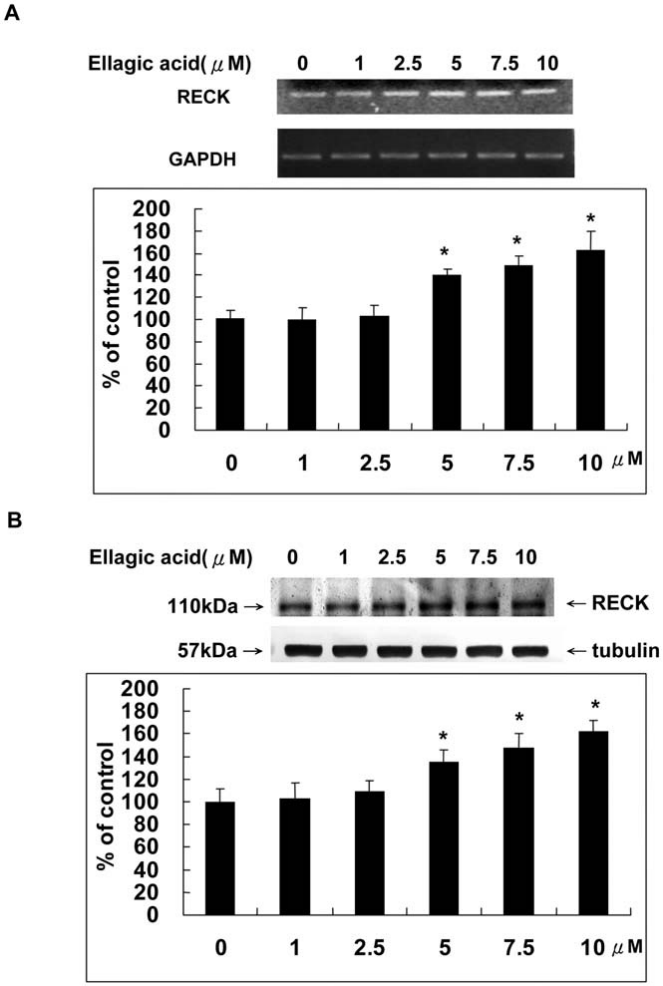
Ellagic acid upregulated the RECK expression. The effect of EA treatment for 24 hours on the expression of RECK in HUVECs was analyzed at the A. mRNA and B. protein levels as measured by RT-PCR and Western blot analysis.

**Figure 6:**
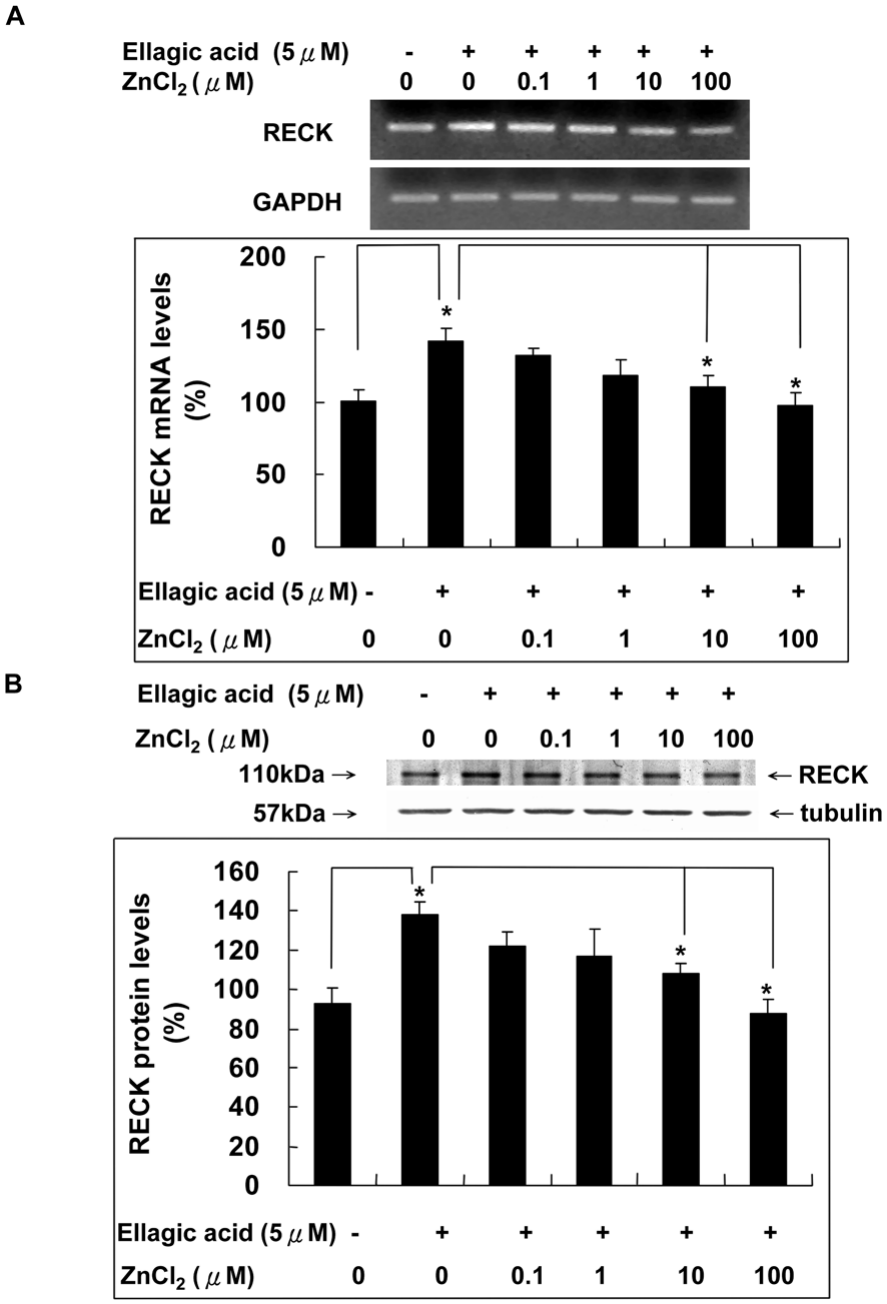
Zinc chloride suppressed the RECK expression enhanced by ellagic acid. The effect of ZnCl_2_ co-incubation with EA on the expression of RECK in HUVECs was analyzed at the A. mRNA and B. protein levels as measured by RT-PCR and Western blot analysis.

RECK is a novel plasma–membrane anchored protein that regulates MMPs activity. RECK inhibits both catalytic and processing steps of proMMP-2 activity [20] and is associated with inhibition of MMP-2, MMP-9 and MMP-14 secretion [24]. Our data showed that both of the mRNA and protein level of RECK were enhanced by EA dose dependently (Fig. 5, A and B). More interestingly, co-incubation with ZnCl_2_ abolished the effect of EA on up-regulating the expression of RECK in HUVECs both at the mRNA and protein levels (Fig. 6, A and B).

### Zinc chelation is involved in the inhibitory effect of ellagic acid on matrix-induced tube formation of human endothelial cells

**Figure 7:**
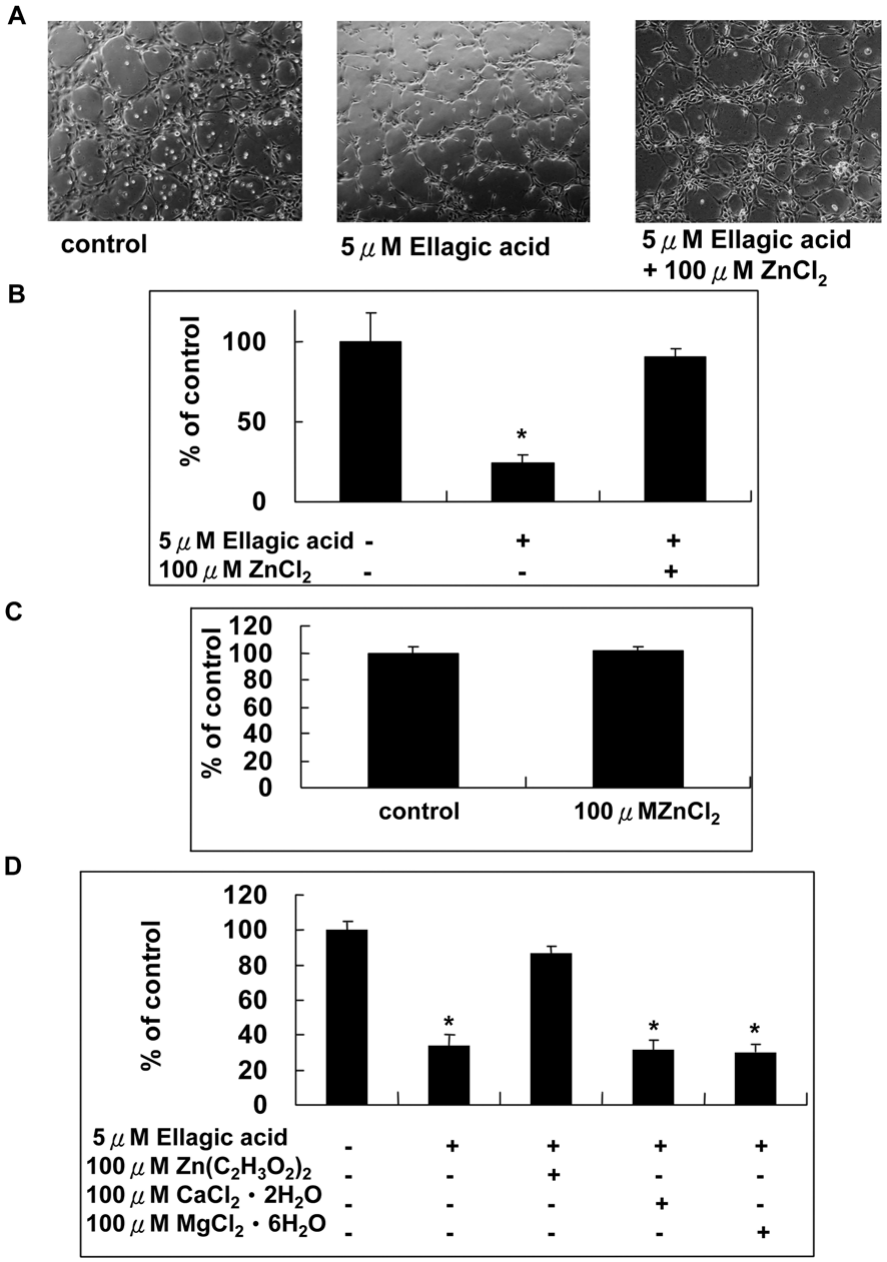
Zinc reversed the matrix-induced tube formation of HUVECs inhibited by EA. A. and B. Cells were treated with 5 µM ellagic acid in the presence of ZnCl_2_ ranged from 0–100 µM for 24 hours. C. Cells were treated with or without 100 µM ZnCl_2_ for 24 hours and processed for tube formation analysis. D. Cells were treated with 5 µM EA in the presence of 100 µM Zn(C_2_H_3_O_2_)2, Ca Cl2 or MgCl2 for 24 hours and processed for tube formation analysis. The number of completely formed tubes in each group was determined and compared. Values are mean (n = 3) ± SEM. An asterisk demonstrated the significant difference (p<0.05) between none-treated and EA-treated groups.

As reported by Labrecque et al. [13], we also demonstrated that that EA ranged from 1–10 µM inhibited the matrix-induced tube formation of HUVECs in a dose-dependent manner (data not shown). Subsequently, we treated human endothelial cells with 5 µM EA in the presence and absence of 100 µM ZnCl_2_ and the tube formation of these cells was compared with control cells. Our result showed that ZnCl_2_ completely reversed the inhibitory effect of EA on matrix-induced tube formation (Fig. 7, A and B). In Fig. 7C, we treated cells with ZnCl_2_ alone and result ruled out the possibility that ZnCl_2_ itself could increase the tube formation of HUVECs. In order to further verify the role of zinc in matrix-induced tube formation, HUVECs were treated 5 µM EA in the presence of 100 µM Zn(C_2_H_3_O_2_)_2_, CaCl_2_ or MgCl_2_. We found that only 100 µM Zn(C_2_H_3_O_2_)2 reversed the tube formation inhibited by EA. Neither CaCl_2_ nor MgCl2 could reverse the inhibitory effect of EA (Fig. 7D). These observations clarified that zinc chelation was responsible for the inhibitory effect of EA on the matrix-induced tube formation of HUVECs.

### Zinc chelation is involved in the inhibitory effect of ellagic acid on migration of human endothelial cells

**Figure 8:**
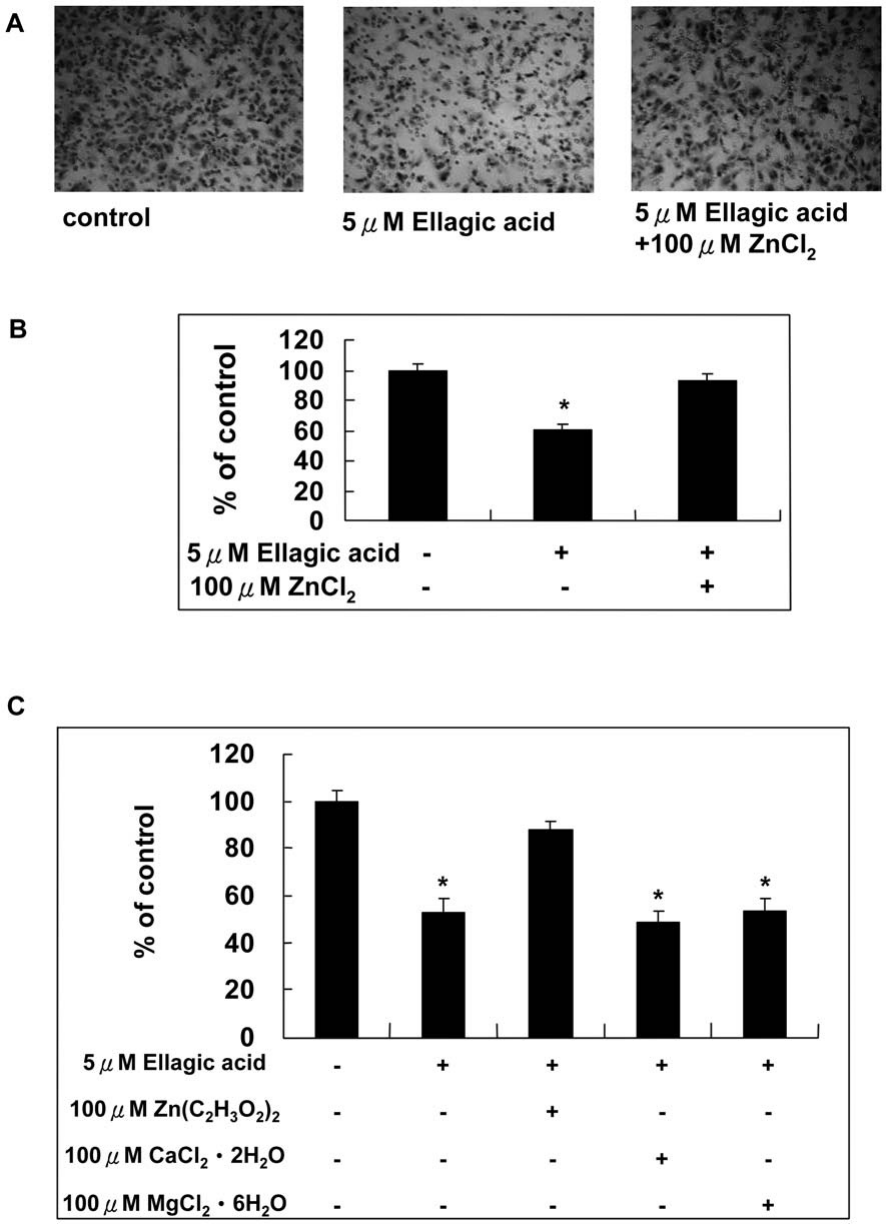
Zinc reversed the migration of HUVECs inhibited by EA. A. and B. Cells were treated with 5 µM EA in the presence of ZnCl_2_ ranged from 0–100 µM for 24 hours. C. Cells were treated with 5 µM EA in the presence of 100 µM Zn(C_2_H_3_O_2_)_2_, Ca Cl2 or MgCl2 for 24 hours and processed for migration assay. Values are mean (n = 3) ± SEM. An asterisk demonstrated the significant difference (p<0.05) between none-treated and EA-treated groups.

In our previous study, we also demonstrated that EA ranged from 1–10 µM dose-dependently inhibited the migration of HUVECs [21]. We then treated HUVECs with 5 µM EA in the presence and absence of 100 µM ZnCl_2_ and the migration of these cells was compared with control cells. Our result showed that ZnCl_2_ completely reversed the inhibitory effect of EA on cell migration (Fig. 8, A and B). In order to further verify the role of zinc in cell migration, HUVECs were treated 5 µM EA in the presence of 100 µM Zn(C_2_H_3_O_2_)_2_, CaCl_2_ or MgCl_2_. We found that only 100 µM Zn(C_2_H_3_O_2_)2 reversed the migration inhibited by EA. Neither CaCl_2_ nor MgCl2 could reverse the inhibitory effect of EA (Fig. 8C). These observations clarified that zinc chelation was involved in the inhibitory effect of EA on the migration of HUVECs.

## Discussion

As reported in our previous study, EA is the active compound in *P. urinaria* that exhibits anti-angiogenic activity and inhibits the secretion of MMP-2 protein from HUVECs [21]. MMP-2, like other MMPs, is a zinc-dependent endopeptidase involved in the degradation of the ECM and plays a role in normal tissue remodeling events such as embryonic development, angiogenesis, tumor migration and wound healing. As zinc is essential for endopeptidase proteolytic capacity to degrade the ECM, compounds with zinc-chelating groups, such as thiol or hydroxamate [25], are often used to inhibit the MMP activity. These inhibitors can chelate zinc ion by imposing a distinct bidentate interaction with zinc and form a stable conformation [26]. Matrix metalloproteinase inhibitors (MMPIs) such as batamastat and marimastat are synthetic, low-molecular weight compound with a collagen-mimicking hydroxamate structure to exert chelation of the zinc ion in the active site of the MMPs and act as competitive MMP-inhibitors and exert anti-angiogenesis effect [27]. Since the direct co-incubation of purified MMP-2 protein with EA inhibited the MMP-2 activity and addition of ZnCl_2_ reversed the effect of EA, the anti-angiogenic effect of EA is at least in part mediated similarly as that of MMPIs by chelating the zinc and directly inhibiting MMP-2 activity. The evidence to show the direct binding of EA to Zn has been suggested by previous reports regarding the anchor of EA to the Zn-coordinated water molecule by its phenol group [22] and the formation of EA-Zn complex [23]. The present study demonstrated the direct binding of EA to MMP-2 protein by measuring the change of UV absorption, confirming the zinc-chelating effect of EA plays an important role in inhibiting the MMP-2 activity directly.

In addition to the direct inhibitory effect on MMP-2 activity mediated by zinc chelation, the treatment of endothelial cells with EA for 24 hours could inhibit the secretion of MMP-2 and therefore further reduced extracellular MMP-2 activity [21]. Interestingly, the inhibition of MMP-2 secretion by EA was also reversible in the presence of ZnCl_2_, indicating a cellular mechanism involved which was also zinc-dependent. The cellular secretory process allows rapid mobilization and utilization of MMP-2 enzymes in the early phase of angiogenesis [28]. Among the regulators reported to be involved in the secretion of MMP-2, RECK was recently found to play an important role in this process. RECK protein was initially discovered by its ability to induce reversion in ras-activated fibroblasts. The key action of RECK is to inhibit MMPs such as MMP-2, MMP-9 and MT-MMP-1. The suppression of RECK expression has been found in the studies of a number of human tumors including colorectal, breast, pancreas, gastric, hepatocellular, prostate, and non-small cell lung carcinoma [29]. Therefore, the up-regulation of the RECK expression is considered potentially as a therapeutic approach to limit cancer angiogenesis and metastasis by suppressing vessel branching via MMPs inhibition. Our results demonstrated that the EA-induced RECK expression was associated with the decrease of MMP-2 secretion, enzymatic activity and inhibition of angiogenesis. We also showed, for the first time, that the EA-induced RECK expression at both transcriptional and translational levels was mediated by withholding of a cellular zinc-dependent pathway, further suggesting the important role of zinc in the process of angiogenesis.

There are eleven zinc-dependent histone deacetylases (HDAC) in humans which modify histones and many non-histone protein substrates. Some substrates of these enzymes include proteins that are known to have a role in cancer growth and angiogenesis [30]. Many of the known HDAC inhibitors (HDACIs) that are in clinical trials also possess a hydroxamate group to chelate the zinc and inhibit HDAC activity [31], [32]. The inhibition of HDAC causes hyperacetylation of histones leading to differentiation, growth arrest and apoptosis of malignant cells, representing a new strategy in cancer therapy. RECK is frequently silenced in aggressive tumor cells by the HADC/SP1 binding in the RECK promoter [33]. HDACIs such as trichostatin A and butyric acid exert anti-angiogenic and anti-metastatic effects both in vitro and in vivo known to be mediated by the up-regulation of RECK that blocks MMP-2 activity [34]. Since the function of HDAC relies on the presence of zinc, the chelation of zinc by EA functions also as a HDACI which abolishes the activity of HDAC and therefore up-regulates the RECK and down-regulates MMP-2 activity. The addition of zinc reverses the effect of EA so that MMP-2 activity remains active when RECK expression is low.

As a potential anti-cancer drug, the zinc-chelatuing effect of ellagic acid may also play a role on preventing the early tumor promotion. EA has been found to exhibit antioxidative activity. Besides scavenging free radicals, antioxidants may inhibit signaling enzymes such as protein kinase C (PKC) that play a crucial role in tumor promotion [35]. Oxidant tumor promoters activate PKC by reacting with zinc-thiolates present within the regulatory domain. EA can inactivate PKC by chelating the zinc and oxidizing the vicinal thiols present within the catalytic domain and block the signal transduction induced by tumor promoters. The redox-mediated inactivation of PKC may, at least in part, be responsible for the antioxidant-induced inhibition of tumor promotion and cell growth. Oxidative stress also represents an important stimulus that widely contributes to tumor angiogenesis mediating the angiogenic switch that can be produced by cancer cells and thus contribute to neoplastic transformation and angiogenesis [36]. We believe that the antioxidative activity of EA may also help to reduce the oxidative stress, particularly those produced from zinc-induced oxidation in the cells which deserved further investigation in the future.

In summary, we found that EA can inhibit the activity and the secretion of MMP-2 in human vascular endothelial cells likely mediated by the induction of RECK expression. EA also inhibits the tube formation and migration of human vascular endothelial cells in a dose-dependent manner. These anti-angiogenic effects caused by EA can all be reversed by the addition of zinc, demonstrating the zinc-chelating activity of ellagic acid and also elucidating the important role of zinc in the process of angiogenesis. Compared with either MMPIs or HDACIs that bind mainly to active zinc-containing domain of specific protein targets, the zinc-chelating effect of EA is much more wide-ranging and therefore may be considered as a more effective anti-angiogenic or anti-cancer drug with greater potential. It is known that EA exhibits multiple effects in a variety of tissues and cells, which may simultaneously trigger various molecular mechanisms that help to increase the effectiveness when it is used to inhibit cancer development or tumor angiogenesis.

## Materials and Methods

### Cell culture

Human endothelial cells were isolated from the vein of human umbilical cords and grown in EGM provided by Clonetics (MD, U.S.A.). Cells were maintained in a humidified atmosphere with 5% CO_2_/95% air at 37°C. Human umbilical vein endothelial cells (HUVECs) were passaged 3–5 times prior to use in experiments. To examine the effect of EA on cell function, cells at 80–90% confluency were treated with 0–10 µM EA for 24 hours.

### MTT assay

Cells with or without ellagic acid treatment were washed once with PBS, followed by adding 1 ml DMEM containing 0.05 mg/ml 3-(4,5-dimethylthiazol-2-yl)-2,5-diphenyltetrazolium bromide (MTT). After incubation at 37°C for 1 h, the media were removed and the formazan crystals in the cells were solubilized in 1 ml DMSO for OD (optical density) reading at 570 nm using a spectrophotometer.

### Zymography analysis

Conditioned medium collected from cultures of HUVECs or purified MMP-2 protein were directly processed for analysis on MMP-2 activity. The effect of EA was tested by incubating EA with conditioned medium or purified MMP-2 protein for 10 minutes at room temperature and processed for zymographic analysis. Samples were loaded onto 10% SDS-PAGE gels in which 1% gelatin (Amersham Life Science,Cleveland,OHIO,USA) was incorporated. After migration, gels were incubated with 2.5% Triton-X 100 twice for 30 min at room temperature, washed for 5 min in TNCA (50 mM Tris pH 7.5, 200 mM NaCl, 5 mM CaCl_2_) and further incubated for 16 hrs in TNCA in a shaking bath at 37°C. Gels were stained for 1 hr in coomassie blue (0.1% coomassie brillant blue R-250, 50% methanol, 10% acetic acid) and destained in 5% methanol/9% acetic acid until proper contrast was achieved. White bands on blue background indicated zones of digestion corresponding to the presence of different MMPs.

### RNA isolation and RT-PCR

Total cellular RNA was isolated by lysis of cells in a guanidinium isothiocyanate buffer, followed by single step phenol-chloroform-isoamyl alcohol extraction procedure modified from that previously described [37]. Briefly, EA treated with or without ZnCl_2_ cells were harvested and lysed in 4 M guanidinium isothiocyanate, 25 mM sodium citrate (pH 7.0), 0.5% sodium sarkosine and 0.1 M ß-mercaptoethanol. Sequentially, 1/10 volume of 2 M sodium acetate (pH 4.0), one volume of phenol and 1/5 volume of chloroform-isoamyl alcohol (49∶1, v∶v) were added to the homogenate. After vigorous vortexing for 30 sec, the solution was centrifuged at 10,000× g for 15 min at 4°C. After removal of the aqueous phase, RNA was precipitated by the addition of 0.5 mL isopropanol. One µg of total RNA was reverse-transcribed into cDNA by incubating with 200 units of reverse-transcriptase in 20 µL of reaction buffer containing 0.25 µg of random primers and 0.8 mM dNTPs at 42°C for 60 min. Two µL of cDNA was used as template for the PCR reaction. PCR was performed in buffer containing 10 mM Tris, pH 8.3, 50 mM KCL, 1.5 mM MgCl_2_, 0.2 mM dNTPs, 1 µM of each primer and 5 units Taq DNA polymerase for 30 cycles of denaturation at 94°C for one min, annealing at 55°C for one min and extension at 72°C for two min. The resulting PCR product was analyzed by EtBr stained 1.5% agarose gel electrophoresis. Sequences for the specific primers used in the PCR are MMP-2 forward primer (5′-GTTTCCATTCCGCTTCCAGG-3′) and reverse primer (5′-TGCCCTTGATGTCATCCTGG-3′); RECK forward primer (5′-AAGTCTTGTATTGTTGGAGGAA-3′) and reverse primer (5′-ACTGATGGTCTTGGAGGC-3′); GAPDH forward primer (5′-TTCATTGACCTCAACTACAT-3′) and reverse primer (5′-GAGGGGCCATCCACAGTCTT-3′).

### Western blotting

Protein concentrations were determined by the Bradford method (Bio-Rad, CA). HUVECs were harvested for protein analysis. Samples with equal amount of proteins were subjected to 10% sodium dodecyl sulfate (SDS) polyacrylamide gel electrophoresis (PAGE) and transferred onto a polyvinylidene difluoride (PVDF) (Millipore, Bedford, MA, USA) membrane. The membrane was incubated at room temperature in blocking solution (1%BSA, 1%goat serum in PBS) for 1 hour, followed by a 2 hour incubation in blocking solution containing an appropriate dilution (1∶1000) of primary antibody, e.g. anti-MMP-2 antibody (NeoMarks, Fremonk, CA, USA) and anti-RECK. After washing, the membrane was incubated in PBS containing goat anti-mouse IgG conjugated with horseradish peroxidase (Sigma, St. Louis, MO, USA) for 1 hour. The membrane was washed and the positive signals were developed with chemiluminescence reagent (Amershan Pharmacia Biotech, Little Chalfont Buckinghamshire, England). Membrane was exposed to Fuji medical X-ray film (Fuji Ltd,Tokyo, Japan) for 30 minutes.

### ECM gel-induced capillary tube formation

The extra cellular matrix (ECM) gel-induced capillary tube formation assay was used as an *in vitro* measurement of angiogenesis. Briefly, 24-well culture plate was coated with 75 µl/well ECM gel (11.34 mg/ml) prepared from Engelbreth Holm-Swarm mouse sarcoma (Sigma) and allowed to stand for 30 minutes at 37°C to form a gel layer. After gel formation, 7
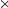
10^4^ HUVECs in 0.5 ml of growth medium were seeded to each well. The plate was incubated at 37°C in a humidified atmosphere with 5% CO_2_/95% air for 4 hours and the formation of capillary tubes were photographed with the use of an inverted microscope.

### Transwell filter migration assay

Transwell filters (Costar, Cambridge, MA) with 8.0 µm pores were used for migration assay. HUVECs were seeded at a density of 1.2×10^5^ cells per filter. To initiate the chemotaxis assay, cells in 250 µl DMEM without FCS were added to the inner chamber and the lower chamber was filled with 600 µl DMEM and 10% FCS as an inducer of cell migration. Cells were allowed to migrate for 2 h at 37°C in an atmosphere of 95% air/5% CO_2_. Cells on the filter were first stained with Liu's stain and cells that remained on the upper surface of the filter were removed using a cotton swab. The cells that migrated onto the lower surface of the filter were examined by microscope after mounting on a slide. Total six random high-power microscopic fields (HPF) (100×) per filter were photographed and the numbers of cells were directly counted.

### Statistical analysis

All statistical analyses were performed using SigmaStat statistical software (version 2.0, Jandel Scientific, CA, U.S.A). Results were represented as means ± STD. Analysis of variance (ANOVA) was carried out when multiple comparisons were evaluated. Values were considered to be significant at *p* less than 0.05.
